# An Active, Multimodal Neural Interface for Real‐Time Monitoring of Cortical Electrical, Thermal, and Optical Dynamics

**DOI:** 10.1002/advs.202512114

**Published:** 2025-10-29

**Authors:** Jiahao Li, Yifei Lu, Zhongzheng Li, Lu Jin, Lianjie Zhou, Ke Ding, Junhan Liu, Bofan Hu, Pengchuan Liu, Dongqi An, Fuying Liang, Yuhang Hu, Yuting Shao, Yifan Ding, Lichao Ma, Rui Li, Yongfeng Mei, Rongjun Zhang, Enming Song

**Affiliations:** ^1^ Institute of Optoelectronics & College of Future Information Technology College of Intelligent Robotics and Advanced Manufacturing Fudan University Shanghai 200433 China; ^2^ Yiwu Research Institute of Fudan University Yiwu Zhejiang 322000 China; ^3^ Shanghai Frontiers Science Research Base of Intelligent Optoelectronics and Perception State Key Laboratory of Integrated Chips and Systems International Institute for Intelligent Nanorobots and Nanosystems Fudan University Shanghai 200438 China; ^4^ Center for Neural Regulation and Brain‐Computer Interface Research Fudan University Shanghai 200438 China; ^5^ State Key Laboratory of Brain Function and Disorders Fudan University Shanghai 200438 China; ^6^ State Key Laboratory of Structural Analysis Optimization and CAE Software for Industrial Equipment School of Mechanics and Aerospace Engineering Dalian University of Technology Dalian 116024 China; ^7^ Department of Ophthalmology Tongji Hospital School of Medicine Tongji University Shanghai 200065 China

**Keywords:** electrocorticograms, multifunctional transistor array, neural interface, photodetectors, temperature sensors

## Abstract

Chronic neurophysiological monitoring devices facilitate the timely diagnosis and treatment of episodic or recurrent neurological disorders. Compared with passive electrodes, silicon‐based active transistors provide intrinsic signal amplification and, when combined with capacitive‐coupling measurement mechanisms, enable high‐density, high‐fidelity recordings. However, most existing systems remain limited to single‐modality electrical sensing and fail to address the growing demands of contemporary neurodynamic research. Here, a chronically implantable, large‐area cortical interface capable of real‐time multimodal monitoring of electrical, thermal, and photodynamic signals is presented. Building upon a silicon‐transistor array for neural electrical detection, the device integrates thin‐film metal resistors for temperature sensing while preserving mechanical flexibility sufficient for stable, long‐term tissue contact. By leveraging the photoelectric effect of silicon transistors and functional multiplexing of active elements, the interface also achieves precise photodynamic measurement. In vitro experiments confirm long‐term stability and channel isolation. In vivo evaluation in Sprague–Dawley rats, together with biocompatibility assessments, demonstrates reliable performance under physiological conditions. The technology used in this multifunctional platform has universal applicability in neural interfaces, offering continuous multimodal neurodynamic data acquisition with potential utility in monitoring, diagnosing, and treating chronic neurological conditions such as epilepsy and brain tumors.

## Graphical abstract

A chronically implantable neural interface enables real‐time multimodal monitoring of electrical, thermal, and photodynamic activities. Incorporating silicon‐based transistor arrays with a functional multiplexing strategy and resistive sensors, the device delivers high‐fidelity recordings with long‐term stability and biocompatibility. This integrated strategy opens new avenues for epilepsy diagnosis and other personalized neuromodulation therapies.

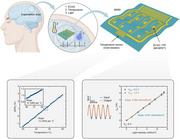

## 1 Introduction

Neurophysiological monitoring serves an irreplaceable role in the diagnosis and treatment of neurological disorders.^[^ ^1^, ^2^, ^3^ ^]^ For example, neural electrical recordings can provide real‐time prevention or early warning of pathological states in chronic conditions such as epilepsy, thereby helping to avoid missing the optimal therapeutic window.^[^ ^4^, ^5^ ^]^ Recent studies also indicate their utility in brain tumor segmentation.^[^ ^6^ ^]^ Conventional clinical neural electrical monitoring primarily employs passive devices, typically conductive metals.^[^ ^7^, ^8^ ^]^ Such devices enable large‐area recording—for instance, silicone‐substrate electrocorticography arrays can cover entire cortical regions,^[^ ^9^ ^]^ and the advent of micro‐ECoG has further improved spatial resolution.^[^ ^10^ ^]^ Passive electrodes, however, inherently lack built‐in amplification, imposing an inevitable trade‐off between spatial resolution (scalability) and signal quality.^[^ ^11^, ^12^ ^]^ To overcome this limitation, neural monitoring has shifted toward active devices over the past decade.^[^ ^13^ ^]^ Active elements such as transistors offer intrinsic signal amplification, enabling both ultrahigh spatial resolution and high signal fidelity, and thus hold great promise to replace traditional passive electrodes in neuroelectrophysiological monitoring.^[^ ^14^ ^]^

Organic transistors, fabricated to thicknesses of only more than a dozen micrometers, exhibit excellent conformability to brain tissue and high signal quality, attracting extensive investigation in neural electrophysiology.^[^ ^15^ ^]^ Nevertheless, their channels are often directly exposed to the biological environment,^[^ ^16^, ^17^ ^]^ leading to interface degradation and performance decline—for example, PEDOT: PSS‐based channels may undergo hydrolysis at the interface, causing signal quality to deteriorate within days.^[^ ^15^ ^]^ Inorganic transistors, which typically use monocrystalline silicon nanomembranes as the semiconductor in flexible electronics, have thicknesses on the order of tens of micrometers and offer faster response rates, making them more suitable for real‐time neural monitoring.^[^ ^14^, ^18^, ^19^, ^20^ ^]^ Unlike Faraday interfaces that directly contact with biological tissue,^[^ ^7^, ^11^, ^18^ ^]^ by employing a capacitive‐coupling strategy with an ultrathin (<2 µm) thermal oxide barrier in contact with biofluids, silicon‐based devices often demonstrate long‐term operational stability,^[^ ^21^ ^]^ with projected lifetimes up to six years, rendering them particularly appropriate for chronic neurological applications requiring months or years of monitoring or therapy.^[^ ^22^ ^]^ Recent work has produced silicon‐transistor arrays exceeding 1000 channels at ultrahigh density (<330 µm) via multiplexed interconnects, further enhancing the spatiotemporal resolution of active devices.^[^ ^23^ ^]^ Although active devices have made significant progress in neural electrical monitoring, diagnosing and treating neurological disorders often demands more than single‐modality monitoring. Conditions such as tumors frequently involve abnormal cortical temperature^[^ ^24^, ^25^, ^26^ ^]^ and some conditions require optogenetic interventions,^[^ ^27^, ^28^, ^29^, ^30^ ^]^ yet integrated monitoring of electrical, thermal, and optical modalities remains scarce and, where reported, is functionally and performance‐wise limited.^[^ ^31^, ^32^, ^33^, ^34^, ^35^ ^]^

In this work, we introduce a chronically implantable multifunctional neural interface system that supports real‐time, continuous, large‐area monitoring of electrical, thermal, and photodynamic activity in cortical regions. The materials and structural design were built upon recent advances in silicon‐based active devices (transistors) and capacitive‐coupling mechanisms for neural electrical detection.^[^ ^21^ ^]^ The system further integrates passive components (metal resistors) for thermal parameter sensing while preserving the mechanical flexibility essential for a long‐term stable interface with neural tissue. By leveraging the photoelectric effect of silicon transistors and functional multiplexing of active elements, the system also enables precise photodynamic measurements, providing a spatially multiplexed multifunctional array solution. In vitro experiments show that the multifunctional system retains the long‐term stability characteristic of capacitive‐coupled inorganic active devices and that its modalities operate without mutual interference. In vivo studies in Sprague–Dawley rat models, together with biocompatibility assessments, indicate that the performance of the system could be suitable for simultaneous long‐term monitoring of multiple brain activities in clinical settings. Such capability may improve quality of life for patients with chronic neurological disorders by providing objective, accurate, real‐time measurements of diverse neurodynamic parameters and by offering potential avenues for diagnosis and therapy.

## 2 Results and Discussion

Electrical activity and thermal dynamics represent fundamental neurophysiological parameters in the cerebral cortex, with demonstrated pathophysiological significance across prevalent neurological disorders, including neoplastic growth and epileptogenesis. Recent advances in photodynamic technology have enabled light energy to serve as a non‐invasive, spatiotemporally precise tool for probing brain function and treating chronic brain disorders. The development of an implantable multi‐modal neural interface achieves concurrent electrocorticographic (ECoG) recording, cortical temperature mapping, and photodynamic modulation, enabling precisely synchronized monitoring of neuro‐electrical, thermal, and optical dynamics in vivo.

### 2.1 Implantable Multifunctional Neural Interface System (MNIS) Compatible with Both Active and Passive Devices

**Figure 1:**
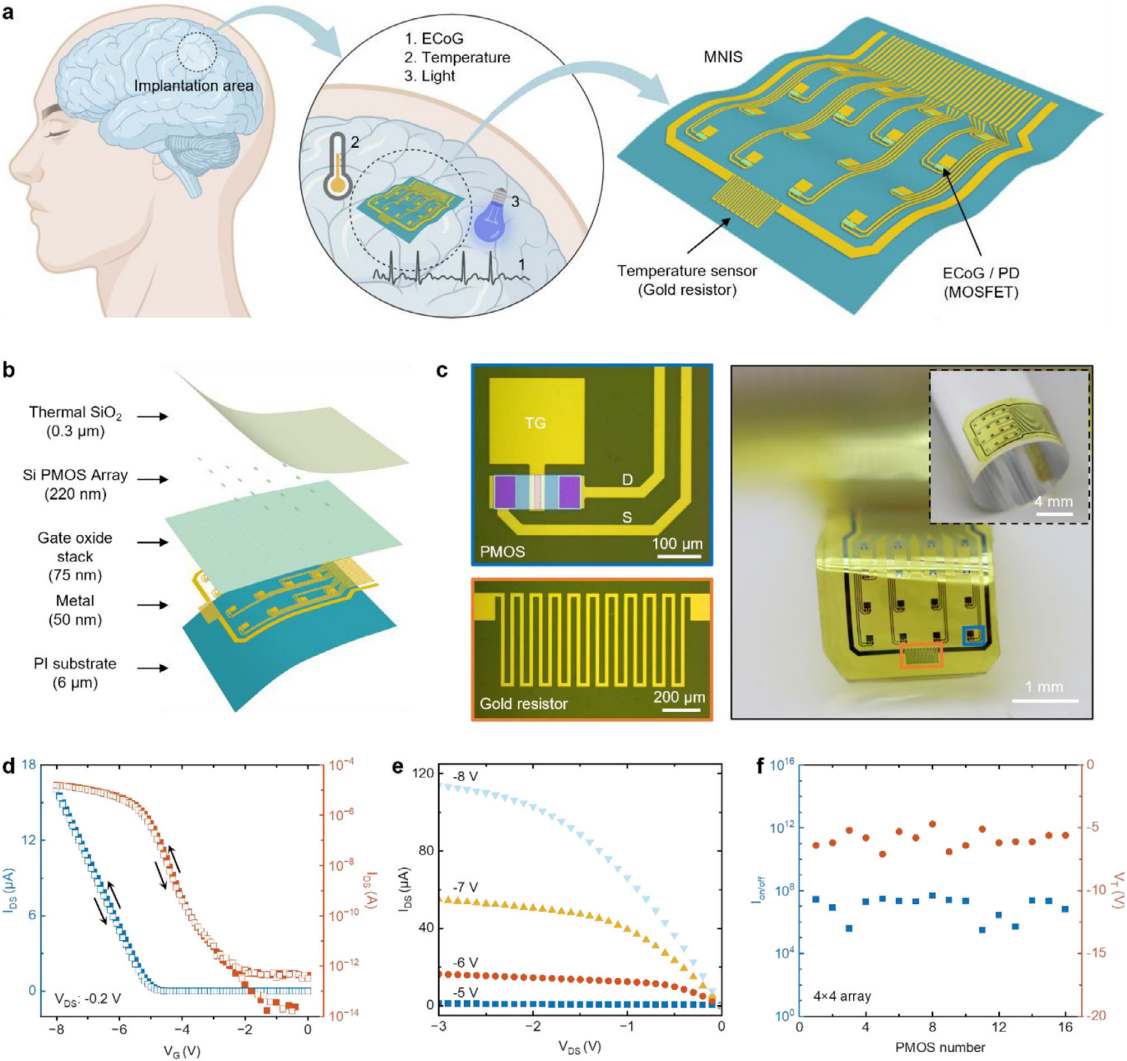
A multifunctional neural interface system (MNIS) compatible with both active and passive devices. a) Schematic of the multifunctional neural interface system for monitoring cerebral cortex signals in ECoG, temperature, and light. ECoG, electrocorticography; PD, photodetection; MOSFET, metal‐oxide‐semiconductor field‐effect transistor. b) An exploded‐view schematic highlighting the key functional layers of the MNIS. c) Photographs of a PMOS for ECoG and PD (top left), a gold resistor for temperature sensing (bottom left), and their integration in the flexible MNIS with 16 PMOS and a gold resistance in a slightly bent state (right). The inset in the top right shows another bending view of the MNIS. PMOS, p‐channel metal‐oxide‐semiconductor; D, drain; S, source; TG, top gate. d,e) The measured transfer (d) and output (e) characteristics of the PMOS in (c). f) Statistics of the on‐off ratio of current (I_on/off_) and threshold voltage (V_T_) of PMOSs from the array in (c).

The integrated MNIS platform that unifies three distinct functional modalities within a flexible implantation region (**Figure** **1**a): 1) high‐resolution cortical electrophysiological recording via ECoG, 2) precise thermal mapping through gold resistive sensors, and 3) optoelectronic detection capabilities. This multifunctional neural interface synergistically integrates active (metal‐oxide‐semiconductor field‐effect transistor, MOSFET) and passive (gold resistor) components, enabling spatiotemporally coordinated in vivo neural monitoring with high performance. Active devices combine dual electrophysiological and optical sensing capabilities, functioning simultaneously as transistor‐based ECoG detectors and diode‐integrated photodetectors (PD). The integrated neural interface comprises a 4 × 4 array of 220 × 300 µm^2^ sensing nodes, uniformly distributed across a 3.28 × 3.36 mm^2^ active area. The exploded‐view schematic of MNIS (Figure 1b) illustrates the key functional hierarchy of the MNIS, consisting of the following layers: from top to bottom, a thermally SiO_2_ layer serving as a bio‐fluid barrier (with a selectable thickness of 0.3–2 µm), a single‐crystal silicon layer (220 nm) forming the p‐channel metal‐oxide‐semiconductor (PMOS) active channel, a gate oxide stack layer of 50 nm SiO_2_ and 15 nm Al_2_O_3_, a metal layer (50 nm Au) serving as interconnects, electrodes, and resistive elements, and a polyimide (PI) layer (6 µm) for encapsulation. Considering the P^+^N junction used in PD, employing PMOS but NMOS can enable single‐step doping that simplifies the MNIS fabrication, and p⁺‐Si exhibits superior stability under physiological conditions.^[^ ^18^ ^]^ To mitigate chronic performance degradation of implanted electrodes, the buried oxide (BOX) layer forms a capacitive interface with biological tissues while simultaneously functioning as a biofluid barrier. The fabrication steps appear in Figure S1 (Supporting Information) and the Experimental Section. The fabrication protocol employs standard semiconductor processing, beginning with direct integration of high‐mobility PMOS transistors on silicon‐on‐insulator (SOI) wafers. Subsequently, a series of photolithography patterns, doping, etching, and deposition formed additional dielectric layers and metal layers for wiring interconnections and a resistor. By employing the SOI backside silicon etching technique, the process thins the silicon layer at the back of the SOI wafer, completely exposing the intermediate BOX layer. This technique allows for complete etching of the bottom silicon layer while preserving the integrity of the silicon layer in the PMOS structure. After Si removal, the device undergoes a structural transition to a flexible, optically transparent architecture. Figure 1c presents the optical image of the flexible device with an overall thickness of ≈31 µm (additionally including a commercial Kapton film used during the backside‐etching step and a polydimethylsiloxane (PDMS) adhesive layer between the Kapton and the PI), captured through the transparent silicon oxide interface (black box, right), with key components detailed in insets: the PMOS transistor architecture (blue panel, top left) and resistive elements (orange panel, bottom left). The inset in the right panel demonstrates conformal adhesion of the device to curved surfaces, confirming its flexibility and compatibility with soft biological tissues. The size of the chip is ≈4 × 4 mm^2^. The top gate electrode area of the PMOS is 220 µm × 200 µm, and the center‐to‐center distance is 1.02 mm, representing the node accuracy and node distance (equivalent to spatial resolution) of the ECoG array, respectively. The PMOS channel occupies a compact 15 µm × 80 µm footprint, matching the photodetector array pitch. This gate‐lead‐free design preserves measurement fidelity while optimizing interconnect density (Figures S1 and S2, Supporting Information). Compared with thermistors and thermocouples,^[^ ^36^, ^37^, ^38^ ^]^ metal resistance‐based temperature sensors, though it has a lower temperature coefficient of resistance (TCR), tend to offer advantages in terms of size and the linearity between resistance and temperature.^[^ ^39^, ^40^ ^]^ The designed gold resistance‐based temperature sensor uses a typical serpentine structure with an area of only 500 µm × 975 µm, suggesting a testing accuracy in space of less than 1 mm. Gold metallization, implemented concurrently with PMOS metal deposition, enhances device yield for both active and passive components while enabling monolithic integration and cost‐effective fabrication. The fabrication and characterization of discrete temperature sensors establishes a critical foundation for developing high‐resolution, flexible implantable sensor arrays.

Representative transfer characteristics (Figure 1d) and output characteristics (Figure 1e) of the PMOS demonstrate that PMOS has a threshold voltage of ≈−5 V, an off‐current lower than 10^−12^ A, an on/off ratio of current (I_on/off_) greater than 10^7^, and good current saturation characteristics, preliminarily confirming the excellent performance of the designed PMOS. From the calculations (Note S1, Supporting Information), the PMOS exhibits a transconductance of ≈5.60 × 10^−6^A/V and an effective mobility of ≈72.9 cm^2^ · V^−1^ · s^−1^. Notably, the flexible PMOS devices exhibit a reduced threshold voltage while maintaining comparable gate leakage to rigid counterparts without back‐etching (Figure S3, Supporting Information), demonstrating that the back‐etching process completely preserves device performance without degradation. Electrical characterization reveals 100% channel yield (I_on_/I_off_ ≥10^5^) across the flexible PMOS array, with uniform threshold voltages (Figure 1f), demonstrating the reliability for scalable production.

### 2.2 In Vitro Assessments of MNIS

**Figure 2:**
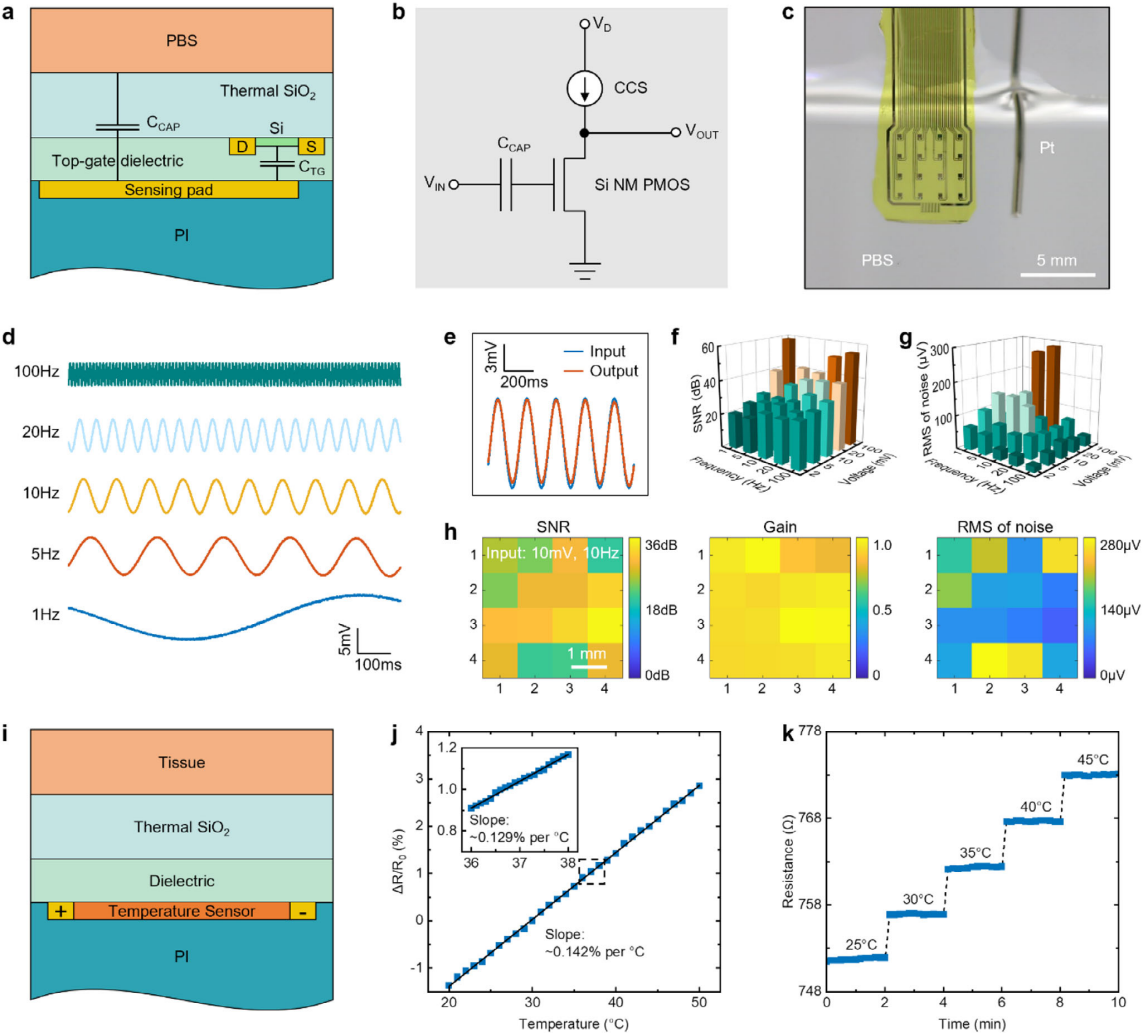
In vitro ECoG and temperature electrical performance assessment. a–c) A schematic of the circuit cross‐section (a) illustrating the mechanism for capacitively coupled sensing through a thermal SiO_2_ layer to an underlying PMOS, its schematic of the circuit from a single‐transistor source following amplifier (b), and its physical prototype image (c) illustrating its in vitro testing environment. C_CAP_, capacitor; C_TG_, top gate capacitance; V_IN_, the voltage of the input a.c. signal; V_OUT_, the voltage of the output a.c. signal; V_D_, the voltage of drain; CCS, constant current source (1µA); Pt, platinum; PBS, phosphate‐buffered saline. d) Output signal characteristics of the PMOS under five different a.c. input frequencies of 10 mV at 1, 5, 10, 20. and 100 Hz, respectively. e) Comparison of input (10 mV, 5 Hz) and output a.c. signals of the PMOS. f,g) 3D histogram illustrating the signal‐to‐noise ratio (SNR) and noise of a typical output signal of PMOS, respectively, along with their relationships to the amplitude and frequency of the input a.c. signal. RMS, root mean square. h) Spatial maps illustrating the SNR, gain, and noise of a typical array's output signal with an a.c. input of 10 mV at 10 Hz. The results indicate a 100% yield, generally high SNR, near‐unity average gain, and relatively low noise. i) A schematic of the circuit cross‐section illustrating the mechanism for sensing temperature through a thermal SiO_2_ layer and a dielectric (50 nm SiO_2_ and 15 nm Al_2_O_3_) layer to an underlying gold resistance‐based temperature sensor. j) Relative resistance change as a function of temperature from 20 to 50 °C, showing a linear variation between 20 and 50°C. The inset shows a measured detail between 36.0 and 38.0°C. k) Resistance as a function of time to demonstrate a small drift in the temperature‐dependent resistance.

The MNIS exhibits stable electrical performance and temperature detection across multiple dynamic ranges in vitro. **Figure** **2**a presents a cross‐sectional schematic of the circuit, illustrating the capacitive coupling mechanism used to sense alternating current (a.c.) signals. The a.c. input signal couples capacitively through both the thermal SiO_2_ layer and top‐gate dielectric to the underlying PMOS transistor. This method significantly suppresses direct current (DC) signal acquisition commonly encountered with other types of sensor modules, such as resistive modules. The schematic of the capacitive coupling device integrated into the external circuit (Figure 2b) shows that the circuit is based on the single‐transistor source‐following amplifier principle. The output signal in the circuit theoretically matches the input signal in both amplitude and frequency, making the input a.c. signal measurable by external devices. Figure 2c depicts the in vitro testing environment, where phosphate‐buffered saline (PBS) solution simulates tissue fluid and the platinum (Pt) needle serves as the a.c. signal input terminal. The thermocompression‐bonded silver wire connects the device's drain–source electrodes to an external circuit. Given that key brain electrical signals, such as delta, theta, alpha, beta, and gamma waves, primarily fall below 100 Hz, the signal frequencies used for this assessment also stay below 100 Hz. Figure 2d displays high‐fidelity output a.c. signal waveforms measured under five representative a.c. input frequencies (1, 5, 10, 20, 100 Hz) with an amplitude of 10 mV, applying a hardware pre‐filtering approach in which a small bypass capacitor is added at the circuit front end to suppress high‐frequency noise (Figure S4a, Supporting Information). This result also shows a low high‐frequency (>300 Hz) noise (that tends to exhibit unneeded spikes) and a decreasing signal amplitude with increasing frequency. Corresponding power spectral density (PSD) analysis further demonstrates that high‐frequency noise exerts only a minimal effect on the output signal (Figure S4b, Supporting Information). However, a noticeable reduction in signal gain at higher frequencies, 100 Hz (0.54), appears compared to that of lower frequencies of 1 Hz (0.99) and 5 Hz (0.93). Figure 2e shows the comparison of input (10 mV, 5 Hz) and output a.c. signals of the PMOS, demonstrating a high fidelity of the signal with a 7% decrease in gain. The 3D histogram in Figure 2f,g explores the effects of a small bypass capacitor on the output signal in signal‐to‐noise ratios (SNRs) and the root mean square (RMS) of noise, under different input frequencies (1, 5, 10, 20, 100 Hz) and amplitudes (2, 5, 10, 20, 100 mV). It shows that increasing the amplitude of the a.c. input effectively improves the SNR, primarily because increasing the amplitude does not significantly affect the noise level, while it noticeably increases the signal amplitude. Additionally, the spatial distribution map in Figure 2h provides a quality assessment of the fabricated PMOS array quality using SNR, gain, and RMS noise metrics, measured at 10 mV a.c. input amplitude and 10 Hz frequency. The array exhibits 87% of devices with SNR >20 dB, peaking above 35 dB (reaching 36.5dB) without using the commercial amplifier and filter. All nodes have a gain between 0.8 and 1, and 75% of nodes exhibit a noise level below 200 µV. It is worth noting that for the higher‐frequency (100–1000Hz) application, forgoing the bypass capacitor pre‐filtering can effectively protect the gain of high frequencies, but the cost is the slight decrease in SNR (Figure S5, Supporting Information). The results above demonstrate reliable multi‐channel signal acquisition with high SNRs across the array.

The circuit cross‐section schematic in Figure 2i depicts the sensing mechanism of the gold resistance‐based temperature sensor embedded in the flexible silicon‐based system. The tested object (in this case, a controlled heating plate) transfers heat through a thin thermal SiO_2_ layer and dielectric layers to the underlying gold resistance. This allows for accurate temperature measurement based on the resistance change induced by temperature fluctuations. Figure 2j shows the relationship between the relative resistance change and temperature within the range of 20–50 °C. The results indicate a high linear correlation between resistance and temperature within the range of 20–50 °C, with a TCR of ≈0.142% per °C, suggesting that the gold resistance temperature sensor has good sensitivity. To verify the temperature sensing accuracy, the inset in Figure 2j shows the relative resistance change within the range of 36–38 °C, measured at intervals of 0.1 °C. The sensor still exhibits a high linear correlation between resistance and temperature and a notable TCR of ≈0.129% per °C, confirming that the sensor is capable of detecting small temperature variations of 0.1 °C. Figure 2k shows the temperature‐dependent resistance change as a function of time, indicating that the sensor exhibits minimal resistance drift at different temperatures (25, 30, 35, 40, 45 °C), contributing to the measurement accuracy during long‐term monitoring. This MNIS demonstrates stable simultaneous recording of neural signals and temperature, combining high‐density integration with reliable long‐term operation for implantable neural applications. The MNIS system uses a single sensor to measure the cortical temperature, considering the wire connections. In future studies, we plan to further miniaturize the temperature sensing element and extend its design toward array‐level integration.

### 2.3 Long‐Term Stability and PD Expansion in MNIS

Long‐term intracranial monitoring places higher demands on the interference resistance and stability characteristics of the system. Capacitive coupling approach employs an insulator layer in contact with the tissues that not only offers the dielectric materials to sense neural electrical activity, but also serves as a barrier to prevent the biofluid penetration to the underlying transistor channels. Such an approach often demonstrates long‐term operational stability with projected lifetimes up to decades,^[^ ^23^ ^]^ rendering them particularly appropriate for chronic neurological applications requiring months or years of monitoring or therapy.^[^ ^22^ ^]^ By comparison, traditional Faraday interfaces are established via electrically‐conductive electrodes (such as gold and platinum), typically relying on direct charge transfer that allows for smaller electrode sizes, higher spatial resolution, and electrical stimulation necessary.^[^ ^7^, ^11^, ^18^ ^]^ However, these electrodes are in direct contact with biological tissues, which means that biofluids tend to penetrate at the biotic/abiotic interfaces, with leakage currents.

**Figure 3:**
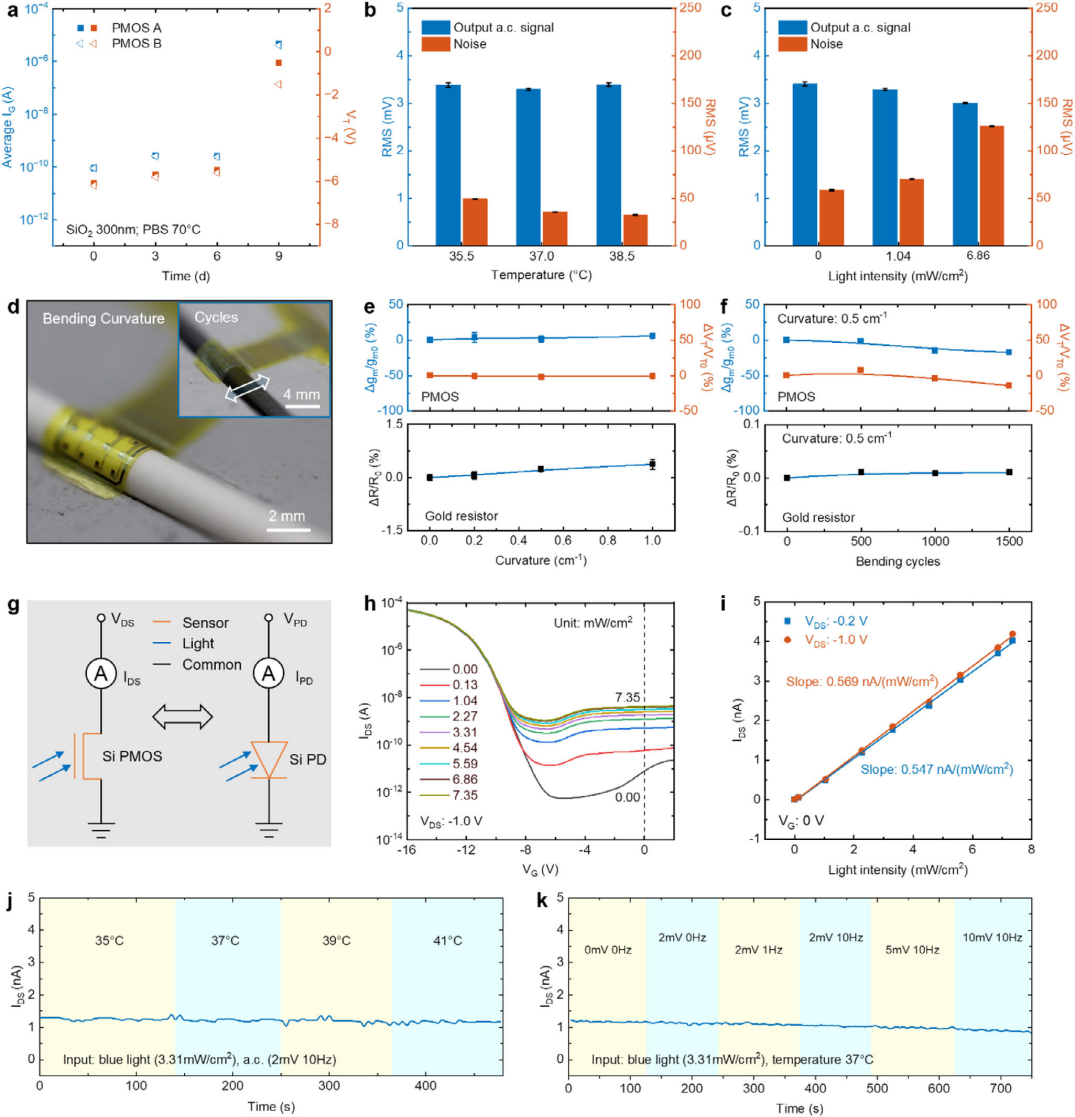
The interference resistance and stability of the system and its expanded PD function assessment. a) Leakage current (I_GS_) and threshold voltage (V_T_) of two representative PMOS devices during PBS soak testing. A small leakage and threshold voltage shifts both appear over a period of 6 days at 70°C using 300 nm thermal SiO_2_. b,c) Histogram illustrating the effects of temperature (b) and blue light (measured at 460 nm) intensity (c) on the RMS values of the a.c. output signal and noise, demonstrating high environmental interference resistance. a.c. input: 10 mV, 10 Hz. d) Images of a flexible chip during a bending test and a bending cycle test (inset). e) Bending characteristics of PMOS (top), and that of the gold resistor (bottom). Relative transconductance and relative threshold voltage of PMOS, and relative resistance of gold resistor as a function of bending curvature. g_m_, transconductance, calculated from the maximum slope. f) Bending cycles characteristics of PMOS (top), and that of the gold resistor (bottom). Relative transconductance and relative threshold voltage of PMOS, and relative resistance of gold resistor as a function of bending cycles. g) Equivalent circuit schematic of the gateless silicon‐based PMOS and silicon‐based photodiode for PD. h) Transfer characteristics of the PMOS under different blue LED intensities (broadband, measured at 460nm). i) Effect of incident light intensity on the drain–source current of the gate‐less transistor under two common drain–source voltages of −0.2 and −1.0 V to illustrate the sensitivity of PD. j,k) The effects of temperature (j) and input a.c. signal (k) on PD, demonstrating a high interference resistance.

Accelerated soak testing using PBS to simulate human tissue fluid has been an effective method for verifying the long‐term performance of implantable devices in a limited time. During the accelerated soak tests, two key parameters of the PMOS—leakage current and threshold voltage—can effectively reflect the degradation of devices (both PMOS and gold resistor). **Figure** **3**a shows accelerated soak tests of two representative PMOS devices (test setup shown in Figure S6a, Supporting Information). Two devices with the thin (300 nm) thermal SiO_2_ bio‐fluid barriers result in small leakage currents and threshold voltage shifts for 6 days at 70°C, with abrupt changes observed on the 9th day. Both devices showed cracks on the 9th day (Figure S6b, Supporting Information), particularly at the leads, primarily due to localized defects leading to leakage, indicating that stringent process control in fabrication could further improve system lifespan. Based on a 300 nm thermal SiO_2_ barrier and its 6 days accelerated soak period, a 300 nm bio‐fluid barrier in PBS at 37°C could last for at least 52 days using Arrhenius modelling (Note S2, Supporting Information).^[^ ^41^, ^42^ ^]^ By increasing the thickness of the thermal SiO_2_ barrier to 1.1 µm, theoretically, the corresponding lifetime of devices will also increase linearly to at least 190 days, which is still reasonable for long‐term applications exceeding six months. However, increasing the thickness of the SiO_2_ layer to enhance long‐term performance may come at the expense of reduced flexibility (Figure S7, Supporting Information). A more prudent strategy would be to tailor the choice of thermal SiO_2_ thickness to the specific application scenario in device design, thereby minimizing unnecessary impacts, such as reduced flexibility, from the oxide thickness.

The interference resistance of the environment is also a challenge for long‐term applications. Figure 3b,c illustrates the effects of temperature and light intensity changes (using a blue LED ring as source—broadband, measured at 460 nm) on detecting a.c. signal by PMOS, analyzed by the RMS of the a.c. output signal and its noise. Clearly, temperature has a limited impact on the output signal RMS and noise RMS, while an increase in blue LED intensity slightly reduces the RMS value of the output signal and increases the RMS of noise (an increment of less than 100 µV) within an acceptable range. Output waveforms (Figure S8, Supporting Information) demonstrate temperature‐ and light‐insensitive a.c. signal fidelity. Similarly, the gold resistance‐based temperature sensor exhibits comparable immunity to variations in ambient light intensity and a.c. interference, with the maximum temperature measurement error remaining within ±0.5 °C (Figure S9, Supporting Information). Figure 3d shows a real chip in the bending test, using a rod with a known diameter to bend the chip. The inset in Figure 3d shows a real chip undergoing mechanical bending cycles, simulating the continuous mechanical fluctuations the system may face in practical applications. The curvature (k = 1/R, where R is the arc radius) change of 1 cm^−1^ has minimal effects on the transconductance and threshold voltage of the three representative PMOS devices (Figure 3e, top). For the gold resistor, a curvature change of 1 cm^−1^ results in about a 3°C error (Figure 3e, bottom). However, considering that the expansion of the cerebral cortex is generally less than 5%, this error has minimal impact when calculating local temperature changes. In the cycles, the PMOS devices maintain high‐quality transfer characteristics even after 1500 cycles of mechanical bending, with only small variations (<15%) in transconductance and threshold voltage (Figure 3f, top). Additionally, the mechanical bending cycles have virtually no impact on the resistance of the gold resistor (Figure 3f, bottom).

In addition to the integration of active and passive devices, the MNIS is also capable of detecting both internal and external parameters. Silicon photodiodes exhibit precise photocurrent‐dependent light intensity detection, as implemented in the circuit for PD (Figure 3g, right). Comprehensive photodiode characterization appears in Figure S10 (Supporting Information), including spectral I‐V behavior, wavelength‐resolved responsivity, intensity‐dependent response, and pulse detection. The P^+^NP^+^ PMOS structure inherently exhibits photodiode‐equivalent operation at zero gate bias, mirroring P^+^N photodiode characteristics. Therefore, the PMOS serves a dual function—detecting both a.c. signals and light intensity, making it a highly integrated, dual‐purpose array in the system. In the simulation, monocrystalline silicon exhibits broad‐spectrum absorption characteristics in the visible and near‐UV ranges, with stronger absorption in the blue and green bands (Figure S11a, Supporting Information), and it is worth noting that blue LED light serves as a commonly employed stimulation source in brain photostimulation. Figure 3h shows the effect of varying blue LED intensities (broadband, measured at 460 nm) on the transfer characteristics of the PMOS (V_DS_ = −1.0 V). As the intensity increases, the transfer characteristics shift upward, with the same phenomenon observed under another typical V_DS_ of −0.2 V (Figure S11b, Supporting Information). Figure 3i demonstrates that the drain–source current of the PMOS with no gate voltage exhibits a highly linear relationship with incident LED intensity at two representative drain–source voltages of −0.2 and −1.0 V, indicating that the device possesses a higher sensitivity (≈0.57 nA (mW cm^−^^2^)^−1^) at V_DS_ of −1.0 V than that (≈0.55 nA (mW cm^−^^2^)^−1^) at V_DS_ of −0.2 V in PD. The photocurrent, at the nanoampere level, suggests that the light‐sensitive area is small, mainly depending on the size of the PMOS channel. The measured PD response time and recovery time of the PMOS are less than 506 and 95 ms (Figure S11c, Supporting Information), respectively, mainly limited by the measurement instrument (Keithley 4200A‐SCS Parameter Analyzer) and the low‐level current in PD. The MNIS‐PMOS architecture additionally enables scalable, high‐density photodetection (Figure S12, Supporting Information). It is worth noting that when light enters from the thermal SiO_2_ side of the MNIS, absorption in the visible and near‐UV range can be neglected. However, when an incident occurs from the PI side, the absorption by the PI layer must be taken into account. The effect of light transmittance of PI with different thicknesses (0.65, 0.95, 6.37, 11.03 µm) on the blue LED source shows that a thinner PI layer exhibits higher transmittance (≈90%) across the band, making it suitable for PD application (Figure S13, Supporting Information). The PMOS photodetector maintains stable performance under concurrent temperature fluctuations and a.c. interference. Figure 3j,k shows that the MNIS maintains high PD testing stability under different temperatures (35, 37, 39, 40 °C) and various a.c. amplitudes and frequencies, with no observable current jumps when conditions change. This MNIS device exhibits excellent long‑term stability, and its expanded PD capabilities enable comprehensive monitoring across neuro‐electrical, thermal, and optical dynamics modalities.

### 2.4 In Vivo MNIS Monitoring of Brain Activity

**Figure 4:**
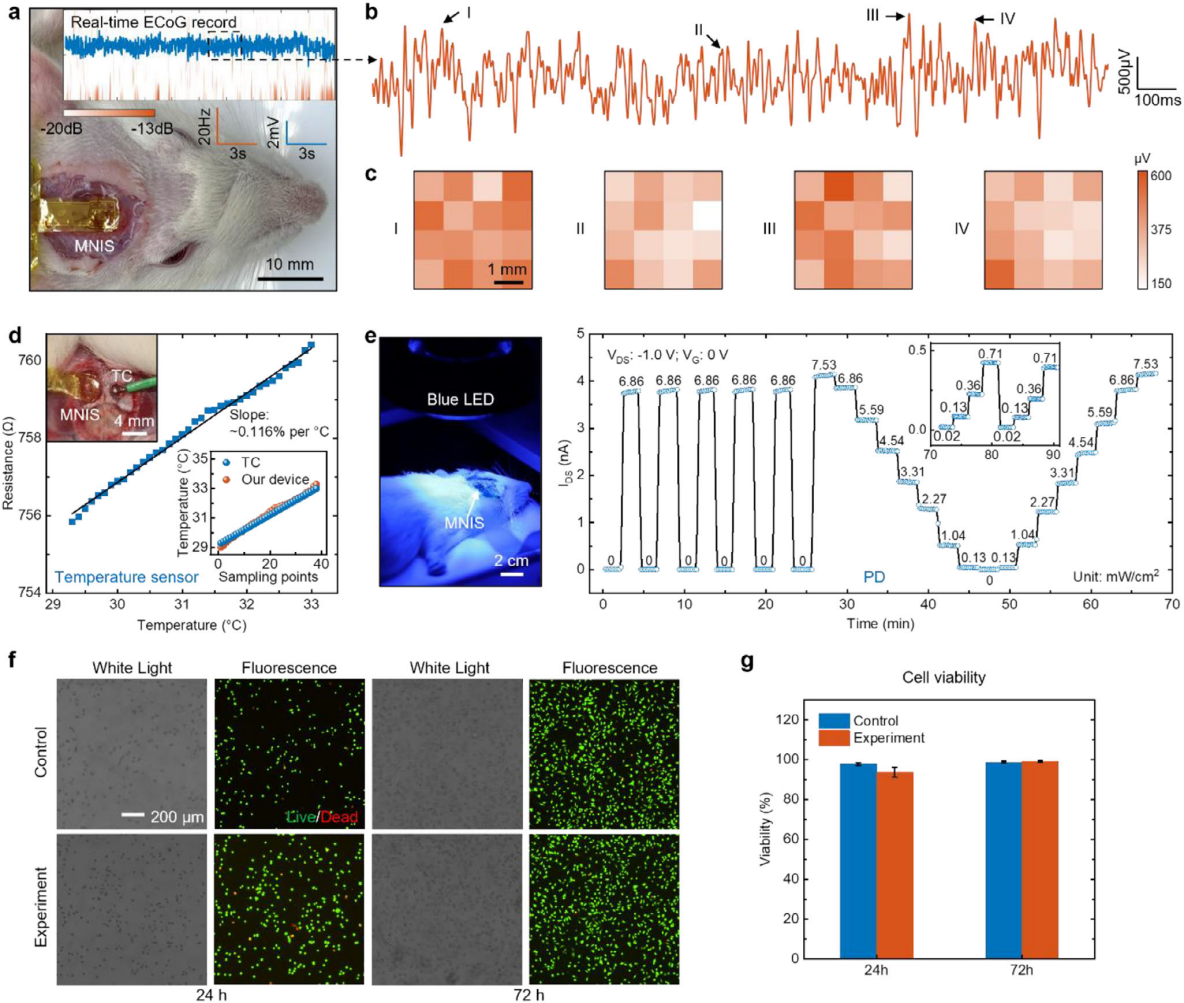
In vivo and biocompatibility experiments. a) In vivo photograph of a partially anesthetized SD rat with MNIS. The inset in the top right shows a real‐time ECoG record from a representative channel and its time‐frequency map. b) A locally amplified ECoG signal from (a) to show the details of peaks. c) The RMS array of four representative sharp peaks (I–IV) from (b). d) The in vivo comparison of the gold resistance‐based temperature sensor and commercial thermocouple (TC) in temperature sensing. The resistance of the gold resistor as a function of the temperature of TC. The inset in the top left shows the in vivo experimental setup for temperature sensing. e) In vivo experimental setup for PD (left) and real‐time detection in light pulse, repeatability under different light intensities. The inset: the PD capabilities in low‐intensity blue LED. f,g) Cell compatibility experiments conducted using L929 cells, comparing cell bright‐field/fluorescence images at 24 and 72 h (f), and a histogram (g) displaying the comparison of cell viability at 24 and 72 h, including both control and experimental groups.

To verify the practicality of MNIS in monitoring ECoG, temperature, and light in the brain cortex, in vivo evaluation in rodents is essential. **Figure** **4**a shows the in vivo experimental setup for ECoG and the real‐time ECoG signal acquisition from a representative channel. The exposure area in the cortical region in the right hemisphere of a partially anesthetized SD rat is ≈6 × 6 mm^2^ through craniotomy and dura mater incision, slightly larger than the size of the MNIS. The time‐frequency map (Figure 4a inset) illustrates that the ECoG signals recorded from the SD rat in a semi‐stuporous state are highly active in the sub‐10 Hz band and exhibit almost invisible background noise. Figure 4b shows an ECoG record signal lasting for 2 s from a representative channel to show the details of sharp peaks. The PSD curve of this 2 s signal segment (Figure S14a, Supporting Information) indicates that low‐frequency components remain dominant, with minimal influence from high‐frequency components (>100 Hz). Figure 4c demonstrates an array recording of the ECoG signals in a partially anesthetized SD rat, and the array map shows the RMS values of signals from a 10 ms window before and after the peak of four representative sharp peaks (I–IV) from Figure 4b. All RMS amplitudes of these four peaks range from 150 to 600 µV, and none of the four arrays exhibited distinct alpha and delta wave features, which is possibly attributed to the relatively low spatial density of the array. The time delays associated with the four peaks across the array are all confined within 25 ms (Figure S14b, Supporting Information), reflecting a high level of temporal alignment and synchronization among the recording sites.

To verify the in vivo performance of the temperature sensor, Figure 4d compares the gold resistance‐based temperature sensor and commercial thermocouple (TC) in temperature measurement on the rat brain cortex. The measured resistance shows a clear linear correlation with the temperature readings from the TC, yielding a TCR of ≈0.116% per °C. Using the TCR obtained from earlier in vitro experiments, the temperature calculated from the measured resistance closely matches the TC values, demonstrating the practical effectiveness of the gold resistor in in vivo temperature sensing (Figure 4d, inset in the bottom right).

The left panel of Figure 4e shows the in vivo PD setup of MNIS, with illumination supplied by an external ring‐type blue LED (broadband, measured at 460 nm) to simulate the optical stimulation. The right panel of Figure 4e illustrates the real‐time detection in optical pulse and repeatability under different light intensities. The minimal photocurrent drift observed during the PD test highlights the excellent stability and reproducibility of MNIS in photodetector applications. Furthermore, the device retains reliable photodetection as the light intensity drops by two orders of magnitude (from 7.53 to 0.02 mW cm^−2^), demonstrating its robustness in low‐light environments.

Excellent biocompatibility facilitates long‐term in vivo monitoring capabilities of the system. Long‐term implantation of rigid devices frequently induces stress‐mediated tissue damage, particularly in sensitive brain regions. Given the high flexibility of the MNIS and its requirement to only adhere to the cortical surface, mechanical damage to the brain cortex after implantation can be minimal. The images of the brain cortex taken 7 h after implantation show almost no trace of the MNIS (Figure S15, Supporting Information). In cell compatibility experiments, Figure 4f shows white light and fluorescence optical images, demonstrating that the distribution of live and dead cells in the control group (without the MNIS) and the experimental group (with the MNIS) is highly similar after 72 h of cell culture. The histogram of statistical cell viability (Figure 4g) reveals that the average viability (99.23%) of the experimental groups at 72 h is comparable to that (98.78%) of the control groups, both being slightly higher than their respective average viability at 24 h (93.78% and 97.92%). The MNIS combines operational stability with demonstrated biocompatibility for chronic in vivo functionality.

## 3 Conclusion

This work introduces a highly compatible, flexible multifunctional neural interface design that combines active–passive device integration with a single‐device functional multiplexing strategy. The demonstrated 4 × 4 silicon‐based PMOS array for electrophysiological and light‐intensity detection, together with resistive temperature sensors, shows excellent performance, long‐term stability, and biocompatibility, with no observable interference among modalities. The small footprint of individual elements in the array provides substantial freedom in spatial layout; with the future incorporation of multiplexed interconnect schemes and further miniaturization of the temperature sensing element, it should be possible to implement three or more sensing function arrays within a single device. It should be noted that transistors underlying the SiO_2_ barriers can suffer from ion diffusion, particularly under positive voltages. A recent study, however, shows that a.c. signals only yield negligible effects on the transistor characteristics with SiO_2_ barriers. Relative to ion diffusion, hydrolysis behavior would be the key parameter that leads to in vivo failure. As a comparison, additional SiC or SiON_x_ materials can effectively block the ion diffusion even under a positive voltage bias applied. Future efforts will focus on the development of processing techniques for the transfer printing of SiC or SiON_x_ barriers, in combination with our MNIS system, for the enhancement of the prevention of ion diffusion.^[^ ^43^, ^44^, ^45^, ^46^ ^]^ Overall, these results and methods advance large‐area, multimodal real‐time monitoring technologies for implantable neural interfaces. The design strategy can not only facilitate studies of thermal effects during optical stimulation—thereby aiding optimization of personalized neuromodulation protocols—but also offer new observational dimensions for investigations of other brain disorders, such as epilepsy focus localization, tumor metabolism monitoring, and closed‐loop optogenetic therapy. In combination with deep learning and related approaches,^[^ ^47^, ^48^ ^]^ this research may help shift neuromodulation from an experience‐driven paradigm toward a data‐driven paradigm.

## 4 Experimental Section

### Fabrication of the MNIS

The chip process flow is shown in Figure S1 (Supporting Information). An N‐type silicon‐on‐insulator (SOI) wafer (220 nm device Si/300 nm buried oxide/500 µm handle Si; Soitec) was mechanically thinned to a 200 µm handle layer. Following standard RCA (Radio Corporation of America) cleaning, a 600 nm SiO_2_ layer, deposited at 300 °C via plasma‐enhanced chemical vapor deposition (PECVD), served as the diffusion mask. Doping windows were defined by photolithography and etched in CF_4_/O_2_ (40:1) via reactive ion etching (RIE), with underlying oxide removed in buffered oxide etchant (BOE). Boron predeposition was performed at 960 °C for 15 min in a tube furnace, followed by drive‐in at 1100 °C for 35 min to form p‐type regions. Isolation trenches were patterned by photolithography and etched in SF_6_/O_2_ (15:3) via RIE. A dual‐layer gate dielectric—50 nm SiO_2_ by PECVD (350 °C, 40 s) and 15 nm Al_2_O_3_ by atomic‐layer deposition (ALD, 250 °C)—was then deposited. Photolithography defined vias regions for the drain and source, and BOE opened the regions. A Cr/Au (5 nm/50 nm) metallization was deposited by magnetron sputtering, patterned by wet etch to form gate electrodes, interconnects, and gold resistors. A 6 µm polyimide (PI) overcoat provided electrical encapsulation. To enable device flexibility, a Kapton film with a thickness of 15 µm was laminated on a glass substrate with a thin layer of cured polydimethylsiloxane (PDMS) as a soft adhesive (curing at 110°C for 60 min). Coating a thin layer of PDMS on the Kapton side, putting the chip with the PI side facing down, applying ≈40 kPa of pressure, and curing PDMS at 110°C for 60 min to achieve bonding between Kapton and chip. The bulk Si handle was subsequently removed by inductively coupled plasma reactive ion etching (ICP‐RIE) in SF_6_/O_2_ (30:6), followed by highly selective XeF_2_ vapor etching (Xenon Difluoride Etching System), which preserved the BOX layer. Photolithography then defined areas for forming openings for contact leads by RIE with CF_4_/O_2_ (40:1) and BOE. Finally, a laser‐cutting procedure defined the outer perimeter of the chip, enabling its release from the handling substrate.

### Data Acquisition

The Keithley 2100 6½‐Digit USB Digital Multimeter (Tektronix, USA) displays the resistance of the temperature sensor in real time during the test and records it using a mobile phone. The PXI data acquisition system was connected through two PXI‐6289 data acquisition cards and collects a.c. or ECoG signals from the output terminals of 16 PMOS devices. A LabVIEW software (National Instruments) controlled the PXI acquisition system and displayed the electrical signal obtained by capacitive coupling measurements in real time on the LabVIEW interface. In the PD experiment, a Keithley 4200A‐SCS Parameter Analyzer was used to acquire weak photocurrent signals in real time.

### Signal Processing

Unless otherwise specified, all a.c. or ECoG signal data were processed using MATLAB software (MathWorks) with band‐stop filtering (49–51 Hz, 99–101 Hz, 149–151 Hz, 199–201 Hz, 249–251 Hz, 299–301 Hz) achieved through the Fourier transform and notch filters, along with band‐pass filtering in the range of 0.1–300 Hz. Additionally, the time‐frequency spectrograms of the ECoG signals and the spatial distribution maps of the ECoG array were calculated and displayed with MATLAB. The optical properties of single‐crystalline silicon were simulated using Ansys Lumerical FDTD. The remaining data plots were all generated using ORIGIN software.

### Accelerated Soak Test

The cured PDMS well was placed on the sensing side of the chip, bonding with an adhesive (Kwik‐Sil, World Precision Instruments) to the thermal SiO_2_ layer without covering the lead region. Detailed experimental settings are in Figure S6 (Supporting Information). PBS solution (1×, pH  =  7.2, Sigma–Aldrich) was injected into the PDMS well, and an oven maintained the temperature at 70 °C. Every three days, the sample was taken out to measure the transfer characteristics of the PMOS devices using a Keithley 4200A‐SCS Parameter Analyzer with a probe station, and fresh PBS solution was replenished into the well.

### Animal Experiments

All animal studies were conducted according to the experimental practices and standards approved by the Animal Welfare and Research Ethics Committee at Fudan University (Approval ID: 202504001S). All in vivo experiments were performed on 6‐week‐old (180g‐220g) Sprague–Dawley (SD) male rats. The rats received anesthesia with 10% chloral hydrate, followed by shaving the hair between the ears and on the top of the head. An ophthalmic scissor opened the scalp to expose the skull. A skull drill created an opening on the right side, revealing approximately a 6 × 6 mm^2^ area of the cerebral cortex (Figure S15, Supporting Information). The test device was connected to a printed circuit board (PCB) through thermocompression‐bonded silver wires and was placed directly onto the cortical surface. As the device relied on capacitive coupling, signal transmission was governed by the interfacial capacitance across the tissue, electrolyte, encapsulation layer, and gate. The SiO_2_ encapsulation served as both an insulator and the dielectric medium of the capacitor. An intimate contact between the biological tissue and the device (e.g., through cortical surface tension or moderate mechanical fixation) could effectively enhance signal coupling efficiency, improving signal fidelity. A PXI system monitored and recorded the ECoG signals from the animal in real time. During photoelectric detection, the ring‐shaped LED light source remained external. The device contacted the cortex with its PI side, while the thermally oxidized SiO_2_ side faced the light source. For brain temperature measurement, the cortex was exposed over areas of ≈6 × 6 mm^2^ and 2 × 2 mm^2^ on the left and right sides of the skull for placement of the test device and a commercial thermocouple, respectively. After inducing euthanasia through cervical dislocation following an overdose of anesthesia, a heating plate adjusted the body temperature. Brain temperatures from both hemispheres were simultaneously measured using the test device and the thermocouple.

## Conflict of Interest

The authors declare no conflict of interest.

## Author Contributions

R.Z. and E.S. conceived and coordinated the project. J.L. designed and fabricated the MNIS and performed the in vitro and in vivo tests. Y.L. and Z.L. assisted in the construction of the in vivo testing circuit of ECoG and the craniotomy experiments for animals. L.J. and L.Z. assisted in data analysis. K.D., J.L., B.H., P.L., F.L., and L.M. assisted in the device fabrication and in vitro characterization. D.A. and R.L. assisted in strain simulation. Y.H. and Y.D. assisted in PD characterization. Y.S. and F.L. assisted in the biocompatibility characterization. J.L. wrote the draft. J.L., L.J., L.Z., Y.M., R.Z., and E.S. reviewed and edited the manuscript.

## Supporting information



Supporting Information

## Data Availability

The data that support the findings of this study are available from the corresponding author upon reasonable request.
