# Supplementary material for: An Active, Multimodal Neural Interface for Real‐Time Monitoring of Cortical Electrical, Thermal, and Optical Dynamics
Source: Adv Sci (Weinh). 2025 Oct 29;13(3):e12114. doi: 10.1002/advs.202512114 (PMC12806190; doi:10.1002/advs.202512114)
Supplement: Supplementary file 1 — Supporting Information [file ADVS-13-e12114-s001.docx]

**Supplementary Information**

**An Active, Multimodal Neural Interface for Real-Time Monitoring of Cortical Electrical, Thermal and Optical Dynamics**

*Jiahao Li, Yifei Lu, Zhongzheng Li, Lu Jin, Lianjie Zhou, Ke Ding, Junhan Liu, Bofan Hu, Pengchuan Liu, Dongqi An, Fuying Liang, Yuhang Hu, Yuting Shao,* *Yifan Ding, Lichao Ma, Rui Li, Yongfeng Mei, Rongjun Zhang*, Enming Song**

E-mail: rjzhang@fudan.edu.cn; sem@fudan.edu.cn

**Supplementary Note 1-2**

**Supplementary Figure S1-S15**

**References**

**Supplementary Note 1: The calculation of the effective mobility of the PMOS transistor.**

The calculation of the PMOS transistor mobility in this study can be carried out as the following equation that defines the effective mobility (*μ_eff_*) in the linear regime^[1,2]^:

$$\mu_{eff}=\frac{Lg_{m}}{WC_{OX}V_{DS}}$$

where *C_OX_* is the specific capacitance of the gate stack per unit area, *L* and *W* are the effective channel length and width, $V_{DS}$ is the source-drain voltage, and *g_m_* is the transconductance. Here, *g_m_* can be defined as:

$$g_{m}=\frac{\partial I_{DS}}{\partial V_{GS}}$$

The magnitude of *C_OX_* can be calculated using the following equation: defined as

$$C_{OX}=\frac{\varepsilon_{0}\varepsilon_{r}}{t_{OX}}$$

where *ε_0_*is the permittivity of free space (8.854 × 10^−14^ F/cm), ε_r_ is the relative permittivity, and t_OX_ is the oxide thickness. For 50 nm SiO_2_, ε_r_ = 3.9, C_OX_(SiO_2_) = 6.90 × 10^−8^ F/cm^2^. For 15 nm Al_2_O_3_, ε_r_ = 11, C_OX_(Al_2_O_3_) = 6.50 × 10^−7^ F/cm^2^. As a result,

$$C_{OX}(total)=\frac{1}{\frac{1}{C_{OX}({SiO}_{2})}+\frac{1}{C_{OX}({{Al}_{2}O}_{3})}}$$

Thus, the *μ_eff_* of the fabricated PMOS transistor could be estimated by these calculated value.

**Supplementary Note 2: The equivalent functional lifetime under 37℃ physiological conditions and 70℃ accelerated aging conditions using the Arrhenius model.**

We used Arrhenius extrapolation to estimate the 37 °C equivalent lifetime of a 300 nm thermal SiO₂ barrier from the observed 6 days survival in PBS at 70 °C. In Arrhenius' relationship^[3,4]^, the acceleration factor (AF):

$$AF=exp(\frac{E_{a}}{k_{B}}(\frac{1}{T_{37}}-\frac{1}{T_{70}}))$$

where temperature $T_{70℃}=343.15 K$, $T_{37}=310.15 K$, and Boltzmann constant $k_{B}=8.617\times{10}^{-5} eV\cdot K^{-1}$. Results for activation energy $E_{a}=0.6 eV$ (a representative range $0.6-1.0 eV$): $AF\approx8.66$. And the equivalent 37 °C lifetime:

$$t_{37}=t_{70}\times AF$$

Consequently, the conservative value of $t_{37} \approx52 d$.


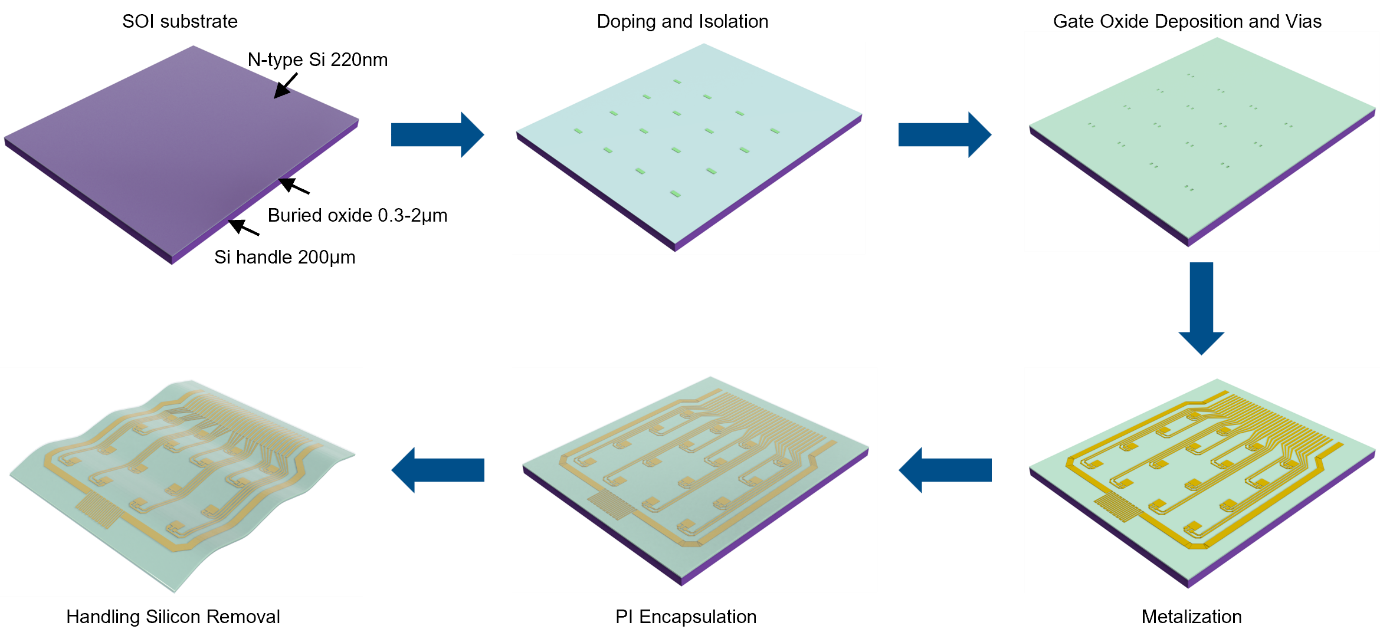


**Figure S1. Chip process flow: from rigid chip to flexible one.**


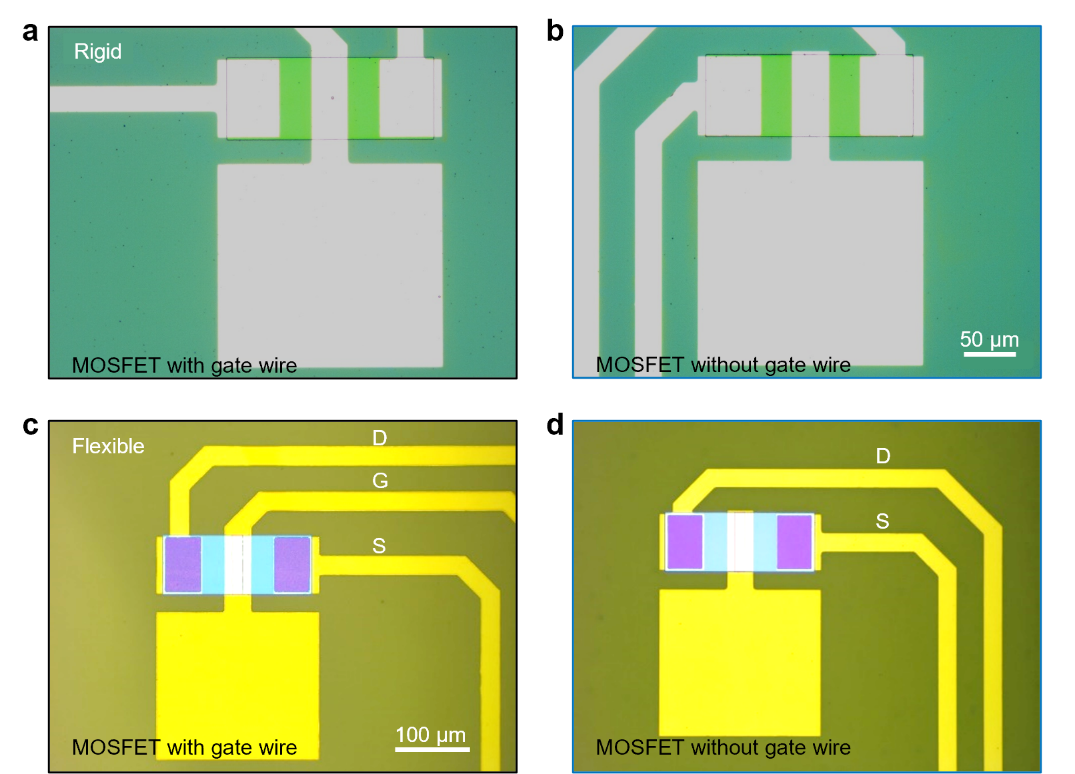


**Figure S2. Optical images of the rigid/flexible PMOS with/without gate wire.**


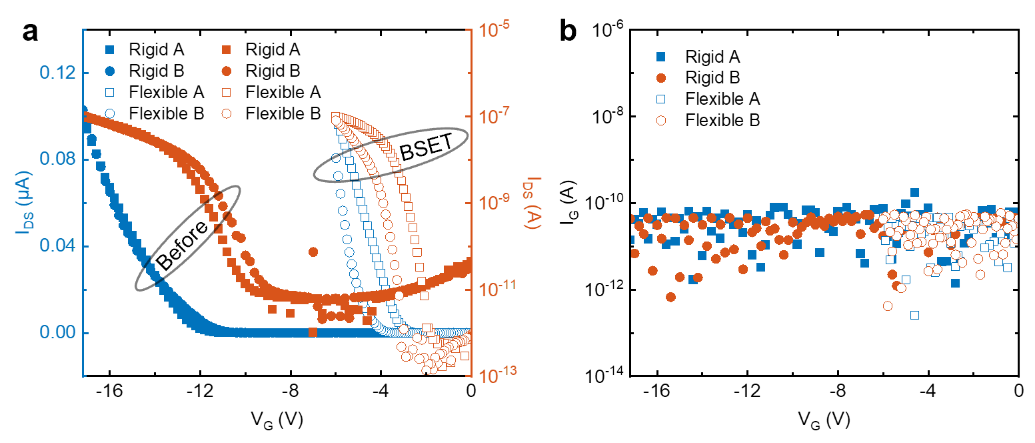


**Figure S3. Comparison of PMOS with gate wire in electrical performance before and after applying backside silicon etching technique (BSET). a**, Comparison of transfer characteristics of the flexible PMOSs with gate wire, compared with those of rigid one. **b**, Comparison of gate leakage current of the flexible PMOSs with gate wire, compared with those of rigid one.


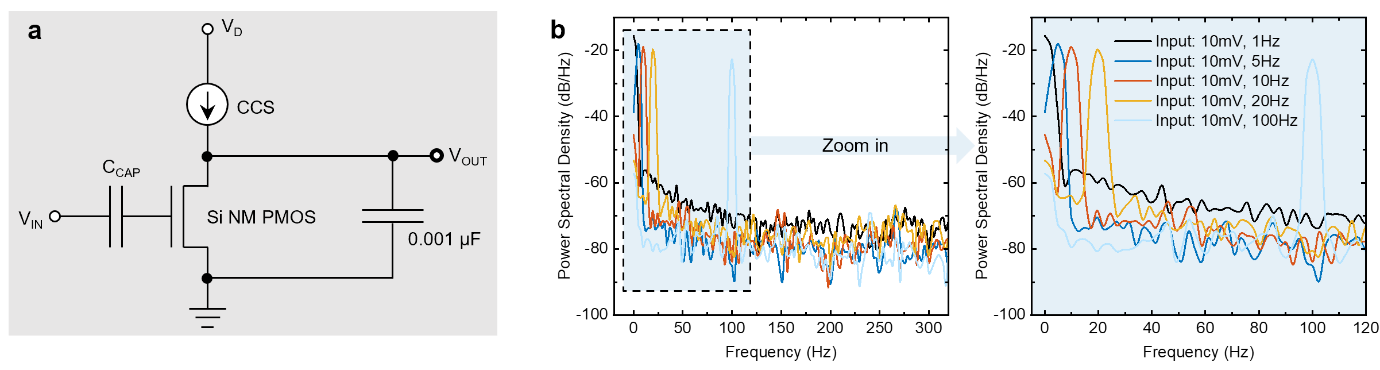


**Figure S4.** **Using bypass capacitors to filter out high-frequency noise. a**, Circuit schematic: Single transistor source follower amplifier with capacitance filter. **b**, In vitro power spectral density (PSD) under five different frequencies (1 Hz, 5 Hz, 10 Hz, 20Hz, 100 Hz) a.c. inputs with an amplitude of 10 mV.


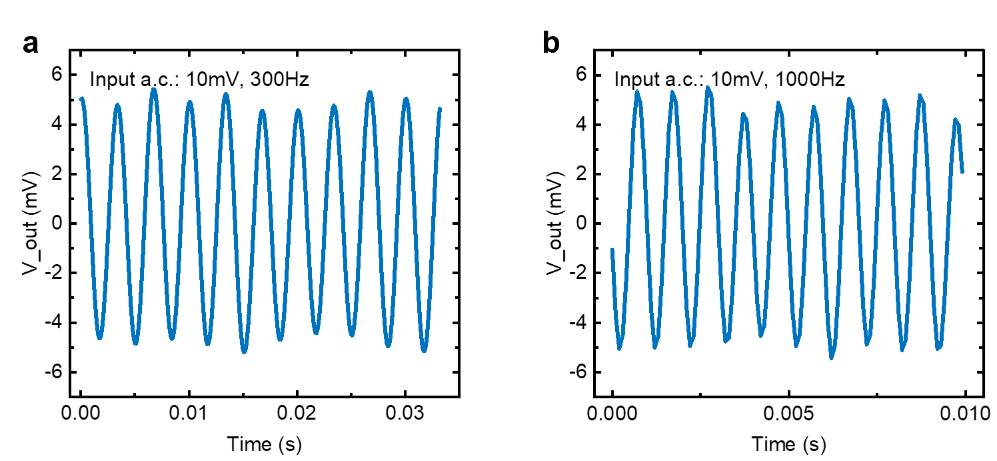


**Figure S5.** **Output performance of high-frequency signals in vitro, forgoing the bypass capacitor pre-filtering. a-b,** Output signal in response to a.c. inputs at 300 Hz (**a**) and 1 kHz (**b**), each with an input amplitude of 10 mV (±5mV). The gains were 0.98 and 1.04, and the signal-to-noise ratios (SNRs) were 21.6 and 21.0, respectively. Power-line interference at 50 Hz and its harmonics were removed.


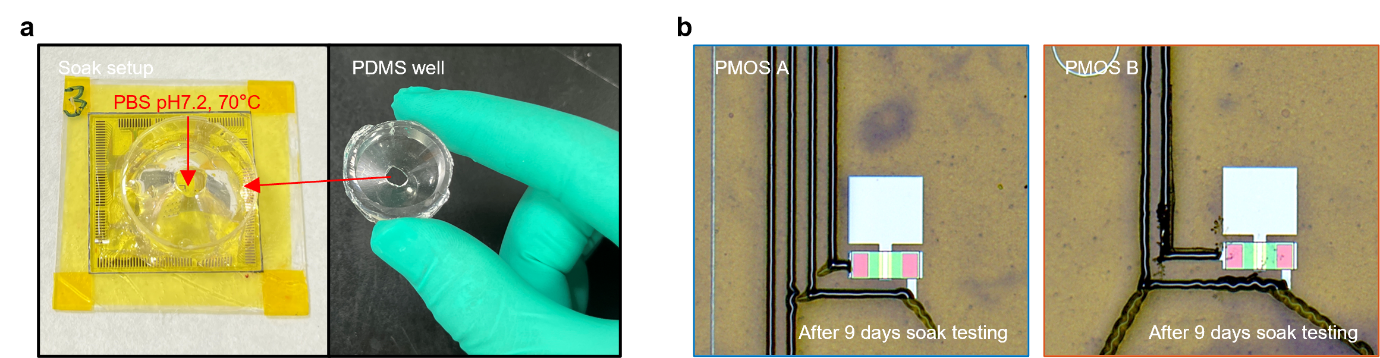


**Figure S6. Accelerated soak test for the chip under 300nm biofluid barrier t-SiO_2_. a**, Experimental setup of accelerated soak test by high temperature Phosphate Buffered Saline (PBS, pH7.2, 70 °C) in a polydimethylsiloxane (PDMS) well. **b**, Images of two representative PMOS after PBS soak test for 9 days.


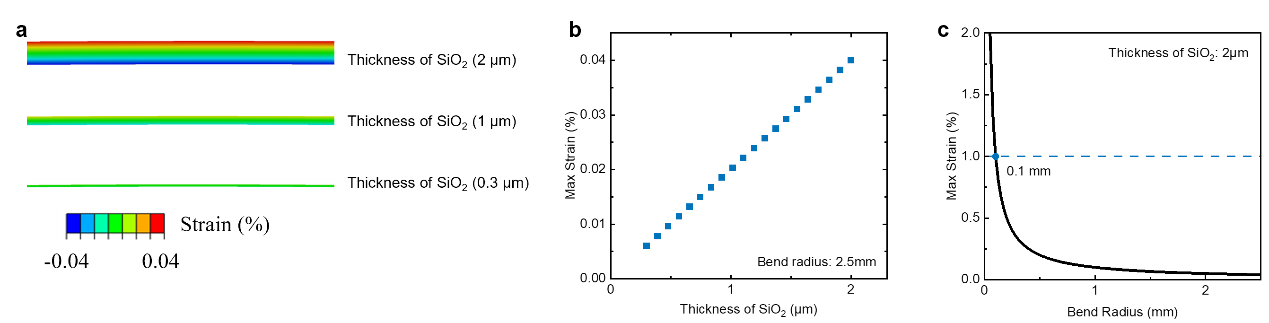


**Figure S7. Effect of layer thickness and bend radius on the strain of the thermal SiO_2_ layer. a**, FEM-simulated strain distribution of the thermal SiO_2_ layer with different SiO_2_ thicknesses under a bend radius of 2.5 mm, showing maximum strains all below the fracture limit (~1%). **b**, Relationship between the maximum strain in the SiO_2_ layer and SiO_2_ thickness at a bend radius of 2.5 mm, where FEM predictions exhibit a consistent linear trend. **c**, FEM prediction of the maximum strain in the SiO_2_ layer with a thickness of 2 μm as a function of bend radius, indicating tolerance to small bend radius (down to 0.1 mm).


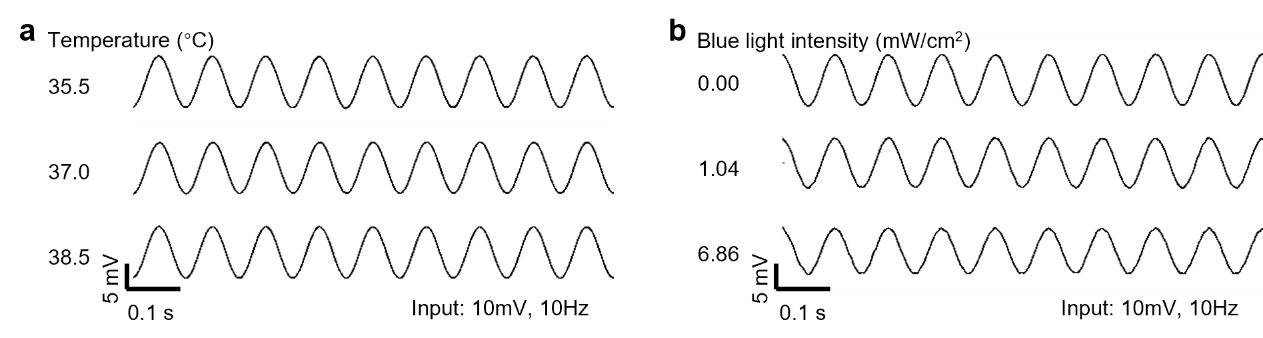


**Figure S8. a.c. signal output characteristics illustrating the environmental interference resistance. a-b**, Output waveform illustrating the effects of temperature (**a**) and blue LED intensity (**b**) on output signal, demonstrating high environmental interference resistance. a.c. input: 10 mV, 10 Hz.


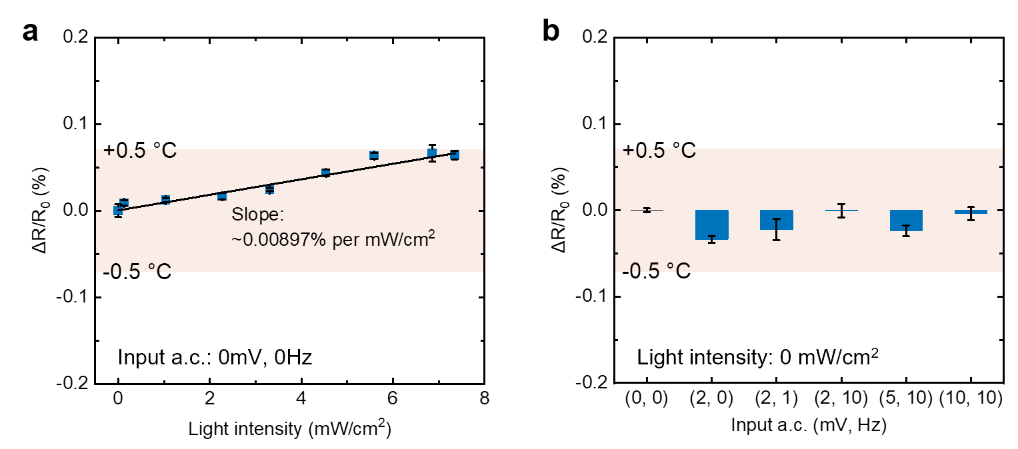


**Figure S9. The interference resistance of gold resistance-based temperature sensor. a-b,** The effects of blue LED intensity (broadband, measured at 460nm) (**a**) and input a.c. signal (**b**) on the gold resistor, demonstrating a high interference resistance of the sensor.


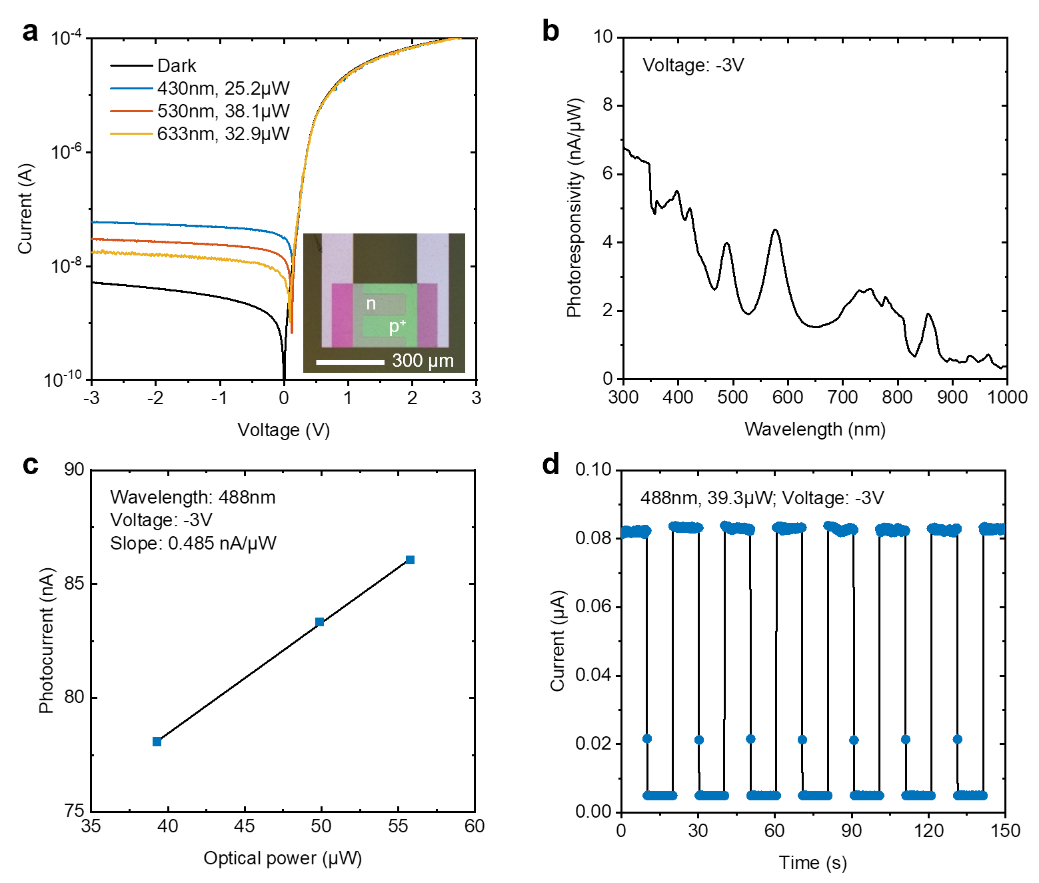


**Figure S10. In vitro silicon-based photodiode performance. a**, Voltage-current characteristics of silicon-based photodiode at different wavelengths. **b**, Wavelength-dependent photoresponsivity. **c**, Current response of the device to varying incident blue light intensity. **d**, Measurement of optical pulses. The light beams above originate from a Xenon lamp source, and the spot area of the used beams is approximately 1 mm².


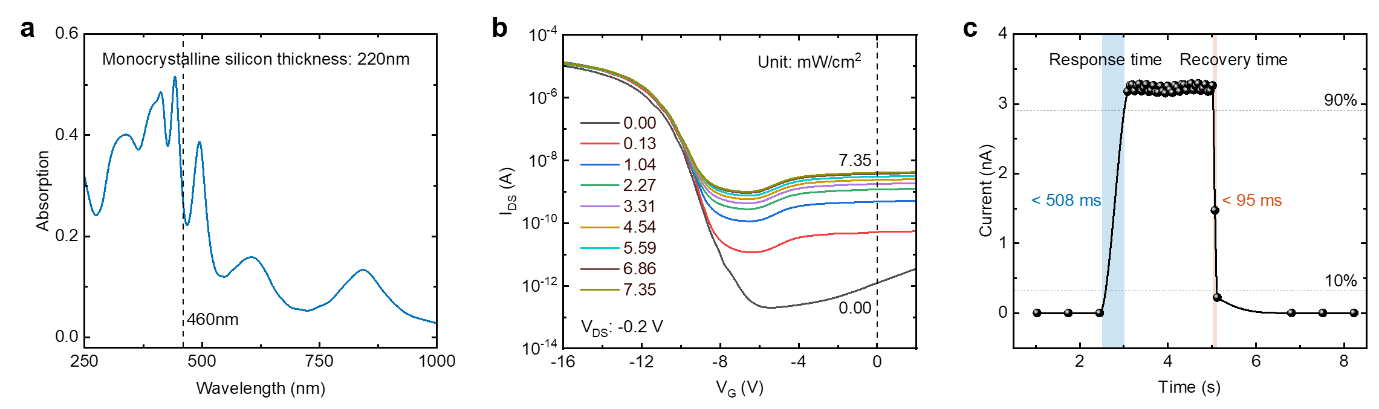


**Figure S11. The photodetectors (PD) characteristics of the PMOS. a**, Light absorption characteristics of 220nm single-crystal silicon, simulated by Ansys Lumerical FDTD, exhibiting broad-spectrum absorption between 250 nm and 1000 nm, especially in the blue band. **b**, Transfer characteristics of PMOS at V_DS_ = -0.2V under different blue LED intensity (broadband, measured at 460nm). **c**, The temporal response of the PMOS for PD, limited by the measurement instrument.


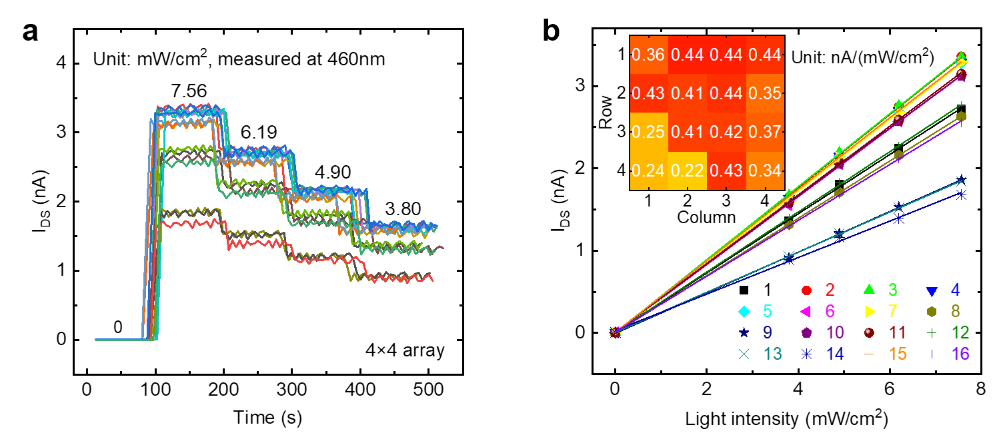


**Figure S12. The photocurrent characteristics of the PD array. a**, Current as a function of time to demonstrate a small drift in the light-dependent PD array. **b**, Linear effect of incident light intensity on the current of the PD array. The Spatial map in the inset shows the photoresponse sensitivity of the PD array. Light source: blue LED (broadband, measured at 460nm).


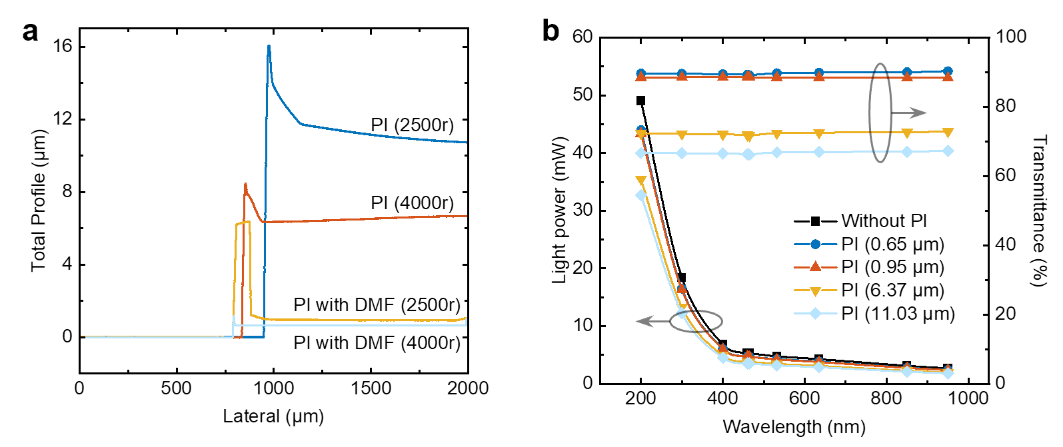


**Figure S13. The effect of the polyimide (PI) protective layer on the incident light source. a**, The influence of spin-coating speed and liquid dilution ratio on the thickness of polyimide (PI). **b**, The spectral characteristics of the blue LED source and the effect of light transmittance of PI with different thicknesses on the source. It shows that thin PI exhibits high transmittance, making it suitable for photodetection.


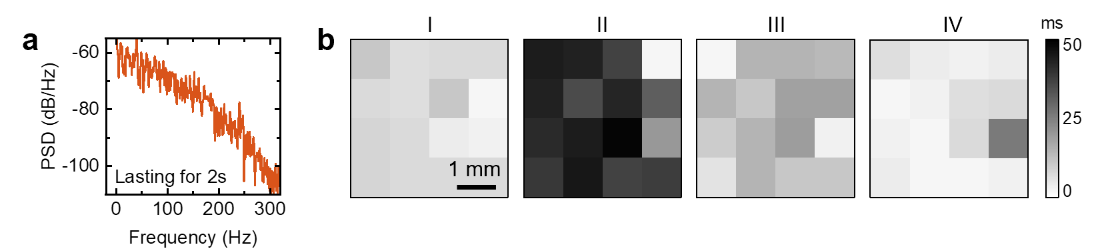


**Figure S14. In vivo experiments. a**, The power spectral density (PSD) of the ECoG signal. **b**, The relative time to four peaks (I, II, III, IV) across the array, demonstrating a high degree of temporal synchronization.


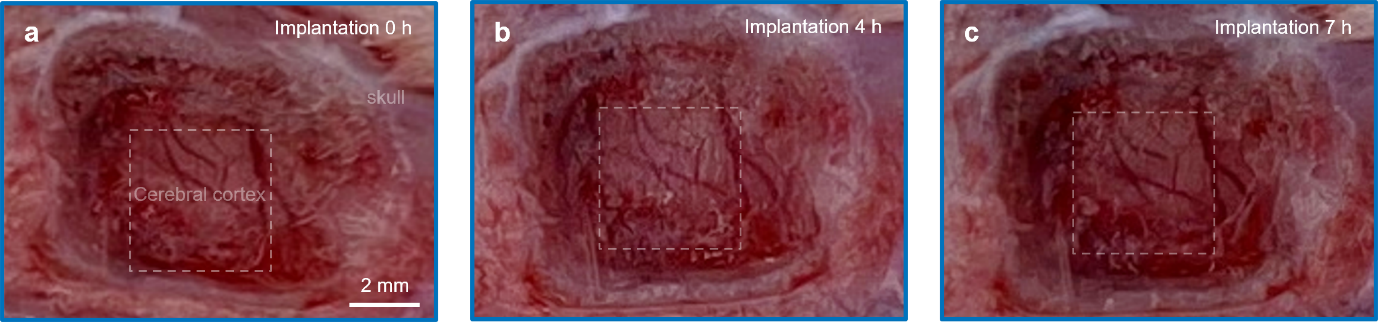


**Figure S15. Mechanical damage test of the brain cortical tissue with flexible adhesive-based devices at different implantation times: The traces of chip implantation are nearly invisible.**

**References**
[1] Fang, Hui, Ki Jun Yu, Christopher Gloschat, et al. "Capacitively coupled arrays of multiplexed flexible silicon transistors for long-term cardiac electrophysiology." *Nature biomedical engineering* 1, no. 3 (2017): 0038. https://doi.org/10.1038/s41551-017-0038

[2] Li, Jinghua, Enming Song, Chia-Han Chiang, et al. "Conductively coupled flexible silicon electronic systems for chronic neural electrophysiology." *Proceedings of the National Academy of Sciences* 115, no. 41 (2018): E9542-E9549. https://doi.org/10.1073/pnas.1813187115

[3] Fang, Hui, Jianing Zhao, Ki Jun Yu, Enming Song, Amir Barati Farimani, Chia-Han Chiang, Xin Jin et al. "Ultrathin, transferred layers of thermally grown silicon dioxide as biofluid barriers for biointegrated flexible electronic systems." *Proceedings of the National Academy of Sciences* 113, no. 42 (2016): 11682-11687. https://doi.org/10.1073/pnas.1605269113

[4] Song, Enming, Rui Li, Xin Jin, Haina Du, Yuming Huang, Jize Zhang, Yu Xia et al. "Ultrathin trilayer assemblies as long-lived barriers against water and ion penetration in flexible bioelectronic systems." *ACS nano* 12, no. 10 (2018): 10317-10326. https://doi.org/10.1021/acsnano.8b05552
